# Geological evidence for AD 1008 tsunami along the Kachchh coast, Western India: Implications for hazard along the Makran Subduction Zone

**DOI:** 10.1038/s41598-018-35193-x

**Published:** 2018-11-14

**Authors:** S. P. Prizomwala, Drasti Gandhi, Nilesh Bhatt, Wilfried Winkler, M. Ravi Kumar, Nisarg Makwana, Nishith Bhatt

**Affiliations:** 1 Active Tectonics Group, Institute of Seismological Research, Gandhinagar, India; 2 Department of Geology, The M. S. University of Baroda, Vadodara, India; 3 Geological Institute, ETH-Zentrum, CH-8092 Zürich, Switzerland; 4 Department of Geology, M. G. Science Institute, Ahmedabad, India

## Abstract

The 2004 Sumatra-Andaman tsunami emphasized the catastrophic nature of such disasters and exposed our knowledge gap of the historical and palaeo events. In the aftermath of this deadly event, the thrust in palaeotsunami studies was restricted to areas in the Indian Ocean, affected by this tsunami. The northern Arabian Sea, which hosts a similar tsunamigenic source i.e. the Makran Subduction Zone (MSZ), has so far remained ‘*Terra-Incognita*’. Here, for the first time, we report geological evidence of the 1008 AD tsunami, also mentioned as ‘*an enigma*’ in the historical reports, by identifying a >250 km long sand sheet with a landward extent of more than 250 m from the Indian coastline. Detailed sedimentology and geochemistry reveals an offshore origin of this sand sheet, from where it was eroded by a high energy wave and deposited in a supratidal environment. Optical and AMS ^14^C chronology constrains its age of deposition around 1000 AD. The shear size of the sand sheet, laterally and across the coast, along with grain size, a characteristically different provenance, are some of the major indicators, which can be useful in palaeotsunami/palaeostorm deposit distinction. Our report of the AD 1008 event from the Indian coastline, also supports the claim that the Western MSZ, albeit at longer intervals, has experienced major thrust earthquakes (*M*_w_ > 8) in the historical past. The proximity of this sand sheet to the shoreline does not discount the role of extremely unlikely, large storms as its causal mechanism.

## Introduction

The giant trans-oceanic Sumatra-Andaman earthquake (Mw ~ 9.3) on 26^th^ December 2004 and the Tohuku earthquake (*M*_w_ ~ 9.0) on 11^th^ March 2011 resulted in tsunami events, which caused massive destruction^1–3^. Since the 2004 tsunami, research has been on an overdrive for identifying palaeotsunami deposits, reconstruct reoccurrence intervals of mega earthquakes and discriminate the sediments deposited by a tsunami wave from those due to a storm wave^4–10^. This is of paramount importance since it aides in mitigating the catastrophic hazard arising out of tsunamis, along the coastal segments. The knowledge of the existence and extent of the past tsunamis is critical in developing warning scenarios. The identification of past tsunamis along with their frequency and severity is particularly important in the implementation of alert systems, which are a must in rapidly developing coastal areas.

**Figure 1:**
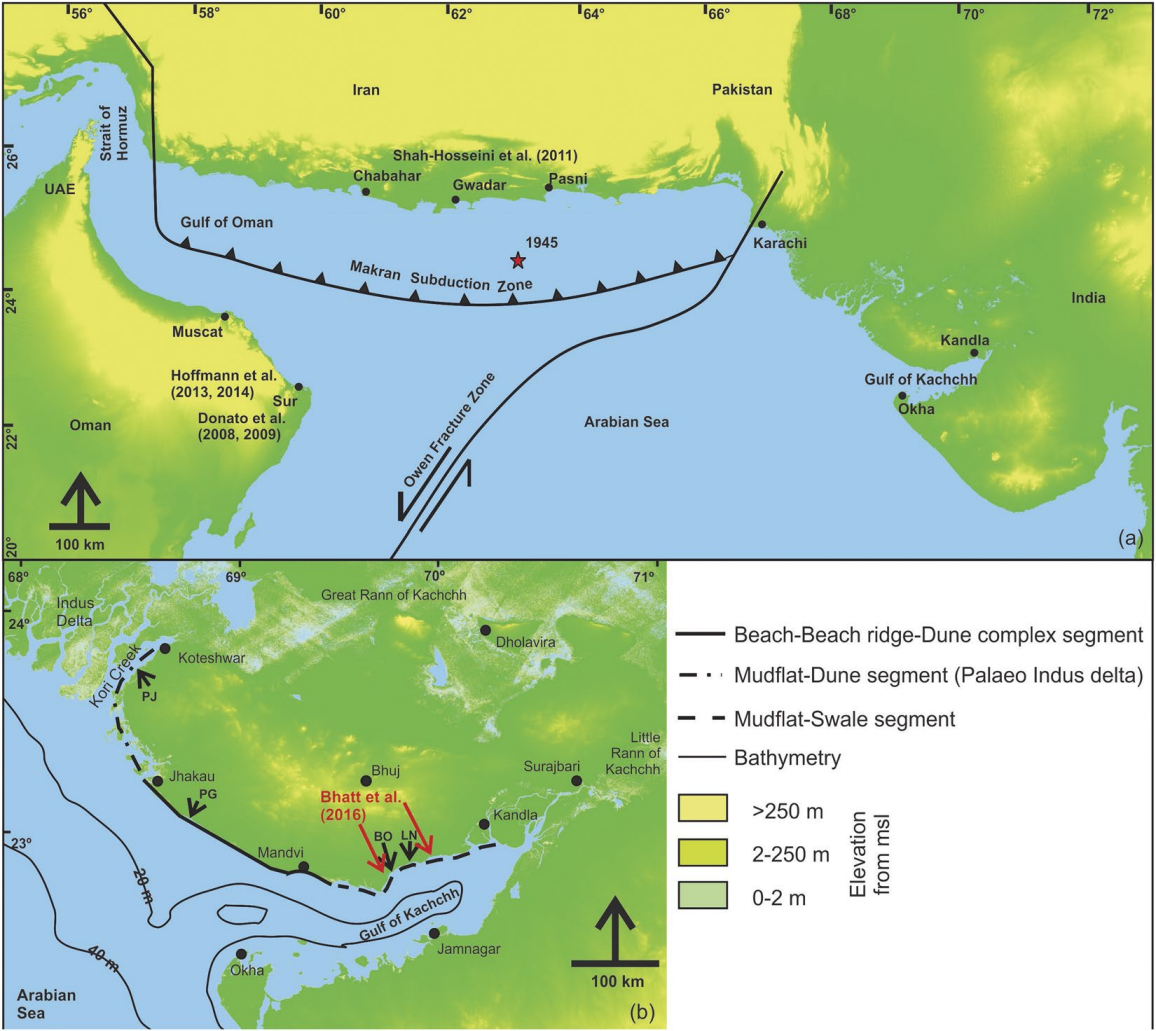
(**a**) Tsunamigenic sources in the Northern Arabian Sea and previously reported tsunamigenic events (**b**) Geomorphic setup of the Kachchh coastline with the studied sites.

Following the advance in research on tsunami and related processes, a number of studies have identified tsunamigenic sources in the Arabian Sea (Fig. 1a), with the Makran Subduction Zone (MSZ) being the most potential source^11^. Based on historical records, several attempts have been made to reconstruct a catalogue of past tsunami events^12–15^. However, it still remains vague and incomplete^9,10^.

The Makran Subduction Zone (MSZ) faces the Oman and Gujarat (India) coastlines in the west and east, as well as the Pakistan and Iranian coastlines in the north (Fig. 1a). Owing to the varied and favorable geomorphic setup^16,17^, the Kachchh coastline in western India, qualifies to be one of the key sites for reconstructing the palaeotsunami history emanating from the MSZ. The wave height along the Kachchh coast due to the last known 1945 tsunami from the MSZ was speculated to be 11 m^18^. However, subsequent geological studies suggested that the wave height is most likely to be an over estimation^9,10^.

The coastline of Kachchh hosts a beach ridge-backswamp configuration from Jakhau to Mandvi (Fig. 1b). It merges with the active mudflats fringed by palaeo-mudflats-swale complexes in the eastern parts from Mandvi to Kandla (Fig. 1b and supplementary data). The westernmost part from Jakhau to Koteshwar shows an intertidal zone fringed by palaeodunes-coastal cliffs (Fig. 1b). Based on OSL chronology and archaeological evidence, the sea level during the 6–3 ka period was inferred to be marginally higher along the Kachchh coast, by at least 1–2 m^19–21^. Bhatt *et al*.^10^ reported a 36 cm thick sand layer sandwiched between mud layers dated 1.3 ± 0.3 ka near Mundra, Southern Kachchh. The extent of this sand layer was about 800 m inland from the present day high water line and spatially about 1 km^2^. The tsunami deposit reported from western India, has a very sharp basal erosional contact, poor sorting, rip-up clasts and mudballs, along with broken coarse shell fragments^10^. On the opposite coastline of the Arabian Sea, Donato *et al*.^22,23^ reported a 5–25 cm thick shell bed in Sur Lagoon along the eastern Oman coastline, which was linked to the 1945 Makran tsunami. The Sur lagoon deposit exhibited a rich taphonomically distinct shell bed with an areal extent >1 km^2^, the provenance of some of the shells being distinctly offshoreward. Similarly, a fine to medium grained poorly sorted sand layer of 40 cm thickness containing shells, corals, wood, debris of bricks, was reported near an archaealogical site of Ras Al-Hadd, Oman^24^. The timing of this event was bracketed to be early Bronze age, i.e. 4450 cal. BP. Based on radiocarbon dating of marine boring bivalves, Shah-hosseini *et al*.^6^ attributed the scattered boulders along the Iranian coastline (northern Arabian Sea) to a palaeotsunami, most likely due to the 1008 AD event in the strait of Hormuz.

In the present study, we report the results from four pits and shallow cores collected from the backswamp and swales along the Kachchh coast fringing the northern Arabian Sea. A sand sheet has been identified based on sedimentology and geochemistry (provenance) for over an extent of >250 km length, which has implications for palaeotsunami research (i.e. distinction of storm and tsunami deposits) worldwide as well as the hazard emanating from the MSZ.

## Geological Evidence of An Extreme Wave Event

The Kachchh coastline facing the northern Arabian Sea in the west and Gulf of Kachchh in the south (Fig. 1a) consists of tide and wave dominating landforms like beach-beach ridge-dune-backswamp complex in the western and wide mudflat-swale dominating segments in the eastern parts^16^. The arid landscape of Kachchh receives an annual rainfall of less than 150 mm, with daily temperatures reaching as high as 50 °C during summer, due to which the coastline of Kachchh is also almost barren. The arid coastal landscape of Kachchh shows that the backswamps in the western and swales in the eastern segment are at an elevation of 2–3 m from the present day high tide line. These are fringed by a dune-ridge of 2–3 m height in front along the seaward side in the West (i.e. Jakhau to Mandvi coastline; Figure in supplementary data) and ridge-runnel system in the East (Mandvi to Kandla coastline). These landforms act as a barrier for the incoming wave as well as provide favorable conditions for preservation of extreme wave deposits by restricting the retreating wave^17^. Also the back swamps in the West and palaeomudflats in the East above the spring high tide levels, act as accommodators during an extreme wave event leading to preservation of the sedimentary deposits^5,17^.

**Figure 2:**
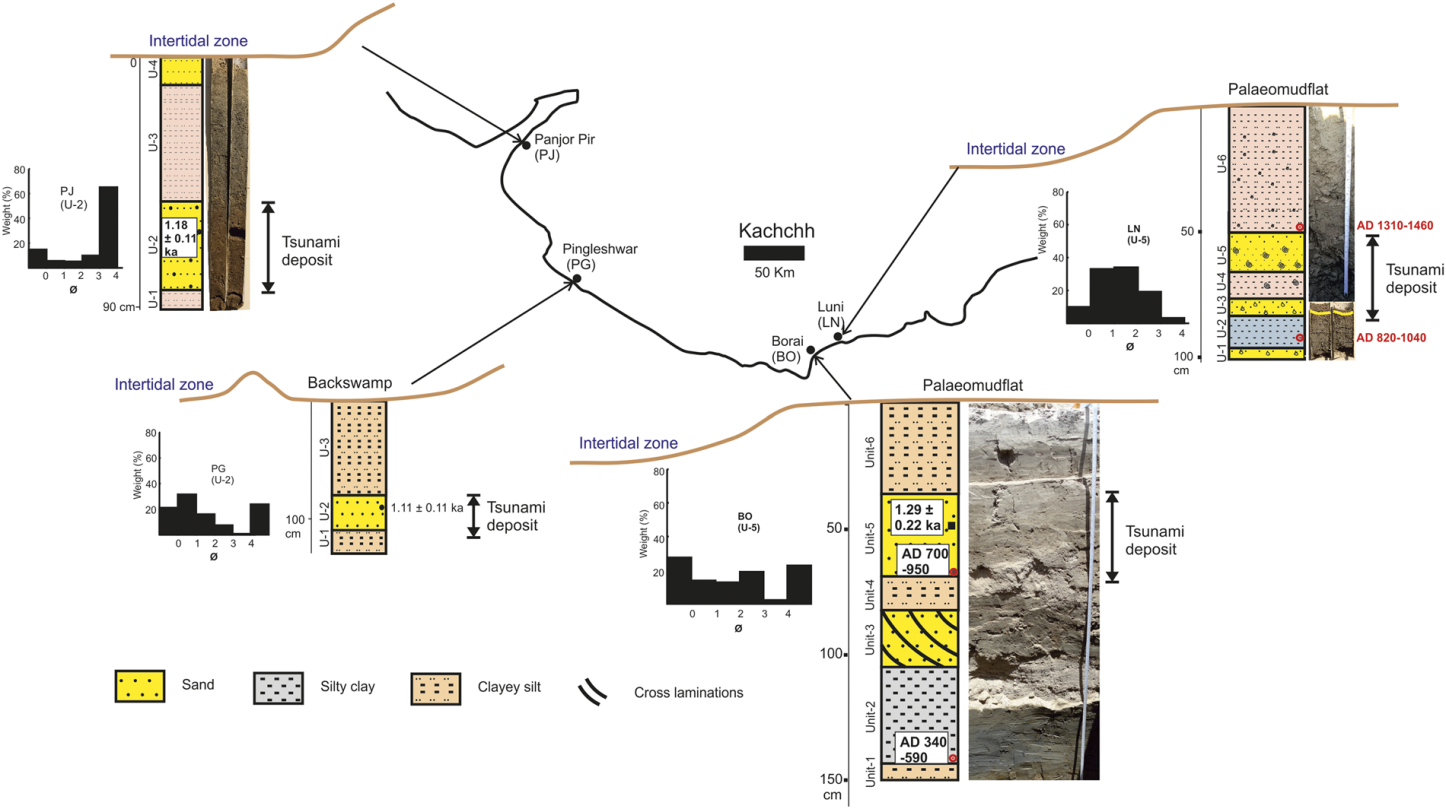
Stratigraphy and photographs of various core/trench sites along the Kachchh coast and the particle size distribution of the tsunami sand layer (Note the bi-model distribution of grain size class in histogram plots).

During the field investigation, we extracted samples from one shallow core (PJ) from the western edge of the Kachchh coast, one shallow pit (PG) from the backswamp of the southwestern edge and two shallow pits from the palaeomudflats (BO and LN) on the eastern edge of the Kachchh coast (Fig. 1b). The 90 cm deep PJ core in a semi enclosed region, i.e. creek (Fig. 2 and Supply Fig. 1a), is located in the present day intertidal flat. The PJ shows a finely laminated clayey silt layer (Unit-1) at the bottom (typical tidal flat environment), which has an abrupt erosional contact with the overlying poorly sorted sand layer (Unit-2) of 32 cm thickness. The grain size distribution shows a bi-model distribution with mean grain size of 2.8 Ǿ (Supply Table 1). This layer shows scattered granules along with mudballs/rip-up clasts present in an assorted manner in the sand horizon. The foraminifers in this horizon show signatures of high energy transport in the form of abrasion marks and broken shells (Dominant Genus: *Ammonia*, *Nonion*, *Pararotalia*, *Quinqueloculina*, *Elphidium*). This unit is overlain by a 42 cm thick dark bluish green colour clayey silt layer (Unit-3) representing a typical mudflat/intertidal environment. The top of the sequence is a fine grained massive sand layer (Unit-4) of 10 cm thickness, representative of a beach-intertidal environment of the present day coastal system.

Another shallow pit PG, dug in the backswamp environment, is at an elevation of 4 m from the present day high tide level in the western Kachchh coastline (Fig. 1b and Supplementary Fig. 1b). The detailed site geomorphology is presented in the supplementary data. The basal unit comprises clayey silt (Unit-1), which showed an erosional contact with an overlying 24 cm thick very poorly sorted coarse grained sand layer (Unit-2). The sand unit shows presence of abundant abraded shells and foraminifers (Dominant Genus: *Ammonia*, *Nonion*, *Pararotalia*, *Quinqueloculina*, *Elphidium*, *Pyrgo*), along with a gravely base. The grain size distribution shows a fine skewed bi-model distribution with a mean grain size of 0.7 Ǿ (Supplemantary Table 1). This sand layer is capped by another clayey silt layer (Unit-3) at the top, with typical intertidal/mudflat sequence characteristics (Fig. 2).

**Figure 3:**
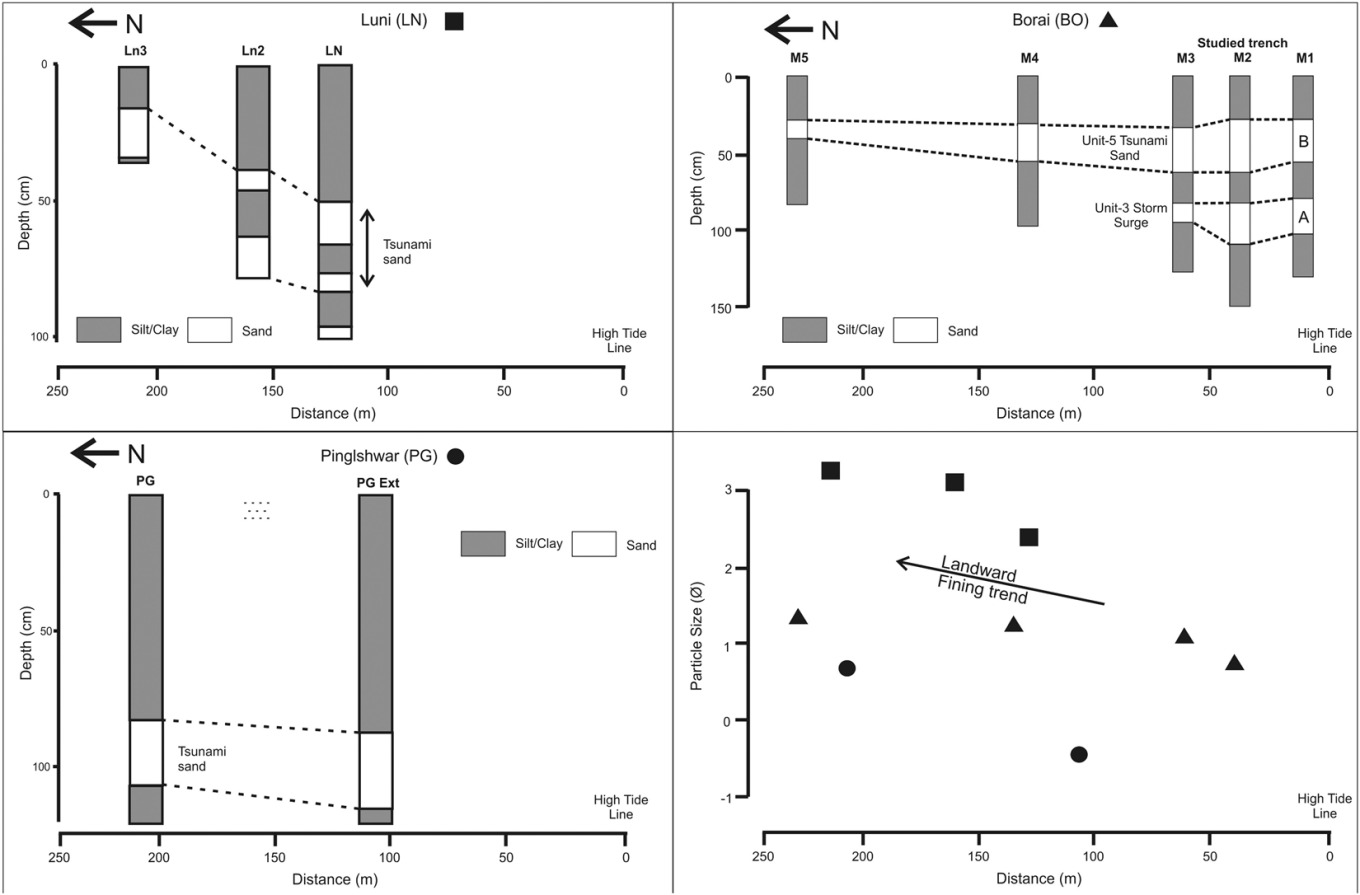
Landward extent and geometry of the sand sheet at various core/trench sites along with landward fining trend based on mean grain size of tsunami sand layer in each trench/core plotted across the coastline.

Similarly, another pit (BO), dug at Borai in a palaeomudflat, is at an elevation of 2 m from the present day high tide line (Fig. 1b and Supplementary Fig. 1c). The detailed geomorphology is presented in Supplementary data. The bottom most unit is clayey silt (Unit-1), overlain by a bluish grey colored laminated silty clay horizon (Unit-2) of 44 cm thickness. Deposition of the clayey unit with laminations in a coastal environment is often reflective of low-energy condition during deposition; a typical feature of mudflat environment. This silty clay unit is overlain by a 24 cm thick seaward dipping mega cross bedded sand layer (A) (Unit-3). The sand is medium to coarse grained, with poor sorting. The seaward dipping orientation of foresets indicates that the deposition occurred during a high-energy current flowing towards the open sea, most likely on account of a storm surge during ebb-tide. This unit is overlain by a clayey silt layer (Unit-4) which is again overlain by a coarse, very poorly sorted 36 cm thick sandy layer B (Unit-5) with a basal erosional contact. The grain size distribution shows a mean grain size of 0.85 Ǿ (Supply Table 1). The presence of mud intraclasts (mudballs) and the abraded nature of foraminifer shells in this sand layer, point to its deposition during a high-energy event, presumably a *tsunami*. The concentration of shells (Dominant Genus: *Ammonia*, *Nonion*, *Pararotalia*, *Quinqueloculina*, *Pyrgo* and Bivalve: *Ensis*) shows an up section increase within this sand unit. Here, the lateral extent of the sand layer B is >250 m from the present day high tide line, whereas, that of the sand layer A is 60 m (Fig. 3).

Another shallow pit LN was dug in a palaeomudflat at an elevation of 2 m from the present day high tide level (Figs 1b and 2). The bottom most layer in shallow core and trench at LN site showed a 4 cm thick coarse grained sand (Unit-1). This layer is overlain by a 13 cm thick bluish grey laminated clay (Unit-2), which is indicative of a tidal flat environment. With an erosional basal contact, a 7 cm thick coarse sand (Unit-3) was resting over the silty clay layer. The sand layer is rich in shells/shell fragments (tests of species: (Dominant Genus: *Ammonia*, *Pararotalia*, *Nonion*, *Quinqueloculina*, *Pyrgo*, *Elphidium*) and shows poor sorting. The grain size distribution shows a coarse skewed distribution with mean grain size of 2.48 Ǿ (Supplementary Table 1). The bottom most part of this unit showed coarser material compared to the top, with a fining upward trend. It is capped by a 11 cm thick organic rich clayey silt layer (Unit-4). Unit-4 reveals scattered broken shells along with patches of sandy material. This unit is again overlain by a 16 cm thick sand layer (Unit-5) which contains mud balls and broken shell fragments. Within this sand layer, the relatively finer sand grains at the bottom show gradation, with coarse grained sand at the top. This unit is also rich in fossils and micaceous minerals, a characteristic product of offshore sediments^16,25,26^. This unit is succeeded by a massive clayey silt layer of 54 cm thickness (Unit-6), which is indicative of regainment of the tidal flat environment.

Similarly, another shallow core from the adjacent Bhadreshwar coast (Fig. 1b), showed a 10 cm thick sand layer, with an erosional, sharp basal contact with the clayey silt unit at the bottom and a graded contact with the overlying clayey silt unit^10^. The sand layer has a coarse grained nature with poor sorting and abundant broken shell fragments.

## Chronology and Provenance

We dated select depth horizons using mollusk shells and foraminifers using the AMS ^14^C technique. Additionally, some preliminary ages have been obtained from optical dating of the sandy horizons at PJ, PG and BO (Table 1). The mollusk shell at the bottom of the sand layer in shallow pits BO and LN bracketed the age of this sand deposition around AD 1000 within the 2-sigma uncertainty levels (bracketed by four ^14^C AMS dates ranging from AD 700 to AD 1460) (Table 2). The optical dating at PJ, PG and BO, suggests deposition of this layer at 1 ka, within 2-sigma uncertainty (Table 1). Earlier, Bhatt *et al*.^10^ reported a sand layer in the Mundra palaeomudflat sequence, dated back to 1.3 ka ± 0.3 ka. The chronological results strongly suggest that the event leading to the deposition of the sand layer at all the sites (barring unit-3 at BO) were deposited around 1 ka BP.

**Table 1:** OSL dating of the tsunami sand sheet at Panjor pir (PJ), Pingleshwar (PG) and Borai (BO) sites.

Sample code	Location	De (Gy)	U (ppm)	Th (ppm)	K (%)	Dose rate (Gy/Ka)	Over Dispersion (%)	Age (Ka)	Reference
ISR-85	Borai	1.9 ± 0.3	1.5 ± 0.07	8.3 ± 0.41	0.7 ± 0.01	1.50 ± 0.11	20	1.29 ± 0.22	This study
ISR-165	Panjor pir	2.03 ± 0.1	1.4 ± 0.07	8.2 ± 0.41	0.99 ± 0.02	1.71 ± 13	36	1.18 ± 0.11	This study
ISR-163	Pingleshwar	1.8 ± 0.12	1.2 ± 0.06	8.6 ± 0.43	0.89 ± 0.02	1.61 ± 0.12	53	1.11 ± 0.11	This study
MU1	Mundra	1.34 ± 0.31	1.25 ± 0.04	6.09 ± 0.29	0.47 ± 0.02	1.04 ± 0.04	—	1.3 ± 0.3	Bhatt *et al*.^10^

**Table 2:** AMS ^14^C chronology from trenches of Borai (BO) and Luni (LN).

Site	Sample code	Material	Depth (cm)	Radiocarbon Age ± 1 sigma (yr BP)	Calendar year ranges (AD) (ΔR = −8 ± 37 corrected^46^, 95.4% or 2 sigma confidence level^45^)
BO (Unit-5)	Bivalve MU/Sh-1	bivalve shell	68	1565 ± 35	700–950 AD
BO (Unit-2)	ETH-46358	foraminifers	149	1930 ± 30	340–590 AD
LN (Unit-6)	Poz-81226	foraminifers	50	950 ± 30	1310–1460 AD
LN (Unit-2)	Poz-81224	foraminifers	95	1445 ± 30	820–1040 AD

**Figure 4:**
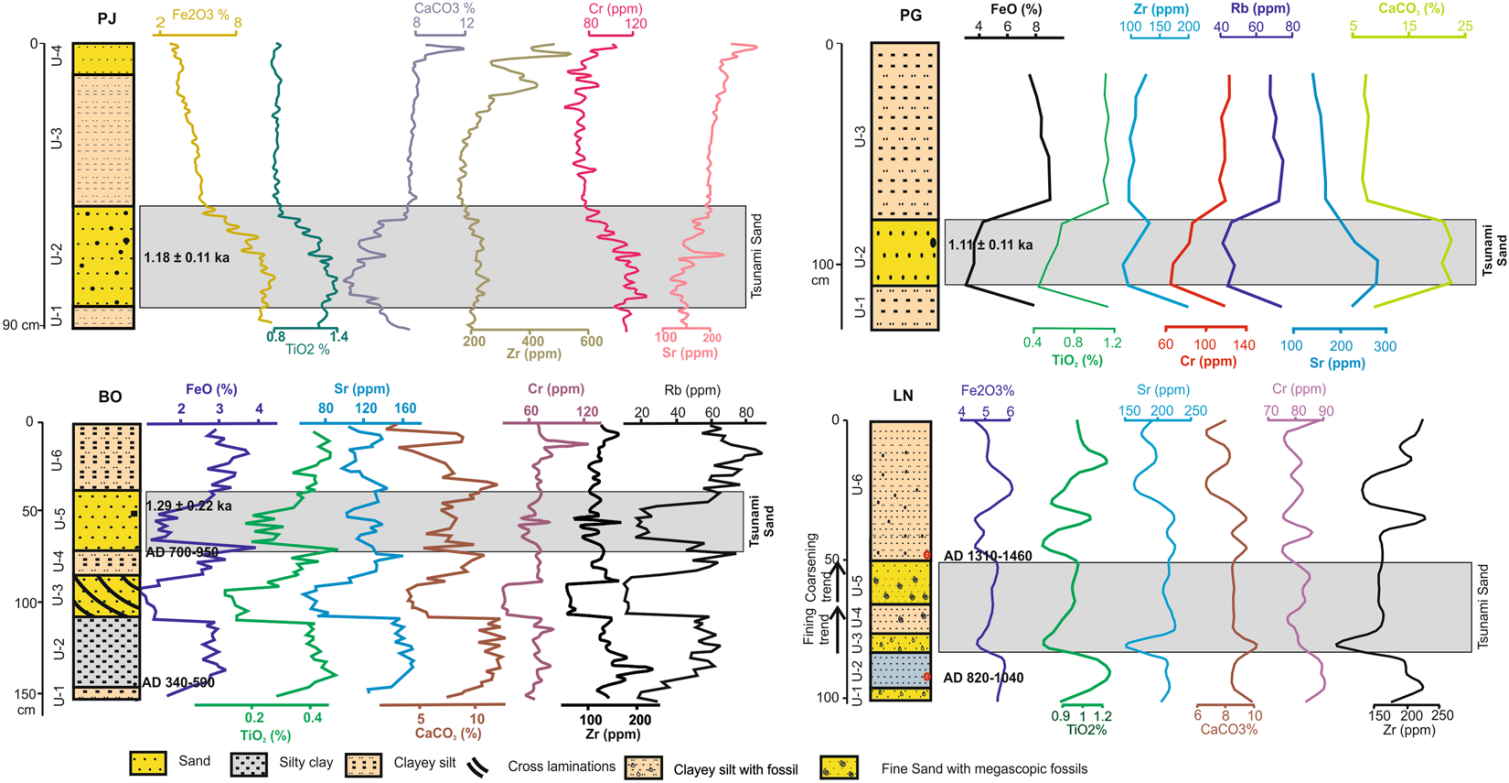
Temporal variations in major and trace elemental geochemistry at main trench/core site from each location.

To further characterize the sand unit, we analyzed samples for specific geochemical indices for tracing the provenance of the sands as well as obtaining hints for the dominance of terrestrial/marine processes (Fig. 4). Earlier works have documented provenance signatures of the coastal sediments and the sediment routing system along the Kachchh coastline^25^. The offshore/subtidal sediments are mostly derived from the Indus and Kachchh Mainland provenance^16,25–27^. The sediments routed from the offshore/subtidal environments are expected to be rich in Zr and Cr^20,21^ along with Sr and sometimes TiO_2_^16,25,27^. This is due to the Kachchh Mainland provenance being rich in Deccan Trap sourced elements i.e. Sr, TiO_2_. The Kachchh Mainland provenance routed from the rivers, mixes with the marine processes in the subtidal environment; where it gets enriched in Zr and Cr during the longshore coastal transport of these sediments. In the present case, the presence of abundant foraminifers and shell fragments is the major cause for the enrichment of CaCO_3_ in the sand layers (Fig. 4). Hence on account of a tsunami event the sand layer would be enriched in Zr, Cr and Sr along with CaCO_3_ if rich in shells (Fig. 4).

The geochemical signatures in the present case, revealed that the sand layer present in pits and cores was routed from the ‘offshore subtidal environment’ onto the respective supratidal regimes (Fig. 4 and see supplementary data for details). This was unambiguous due to the characteristic difference between the hinterland provenance (Deccan dominated^16,25^) and offshoreward provenance (Indus dominated^16,26,27^). Figure 4 shows distinctly different geochemical character of the sand layer compared to the capping mud layers above and below.

## Tsunami or an exceptionally strong Storm

The correlation between different sites showing deposition of the sand layer 1000 years ago, its associated sedimentological and geochemical characteristics, suggest that this event affected a large spatial area (>250 km length) along the Kachchh coast. The physical character of this sand layer viz., its basal erosional/sharp contact with the underlying unit, lack of sorting along with presence of mud-balls and rip-up clasts, abraded foraminifers/shell fragments, bi-modal grain size distribution along with poor to very poor sorting indicates its association with a high energy event, that eroded the substrate of the offshore region and left a chaotic sediment deposit in the supratidal region. Detailed reviews of sedimentologic characteristics of tsunami deposits by Morton *et al*.^28^, Kortekass and Dawson^29^, Shanmugam^30^ and Tuttle *et al*.^31^, support our interpretation: These studies suggest that tsunami sand layers differ from storm deposits due to: (a) presence of mud intraclasts or mud flakes, (b) the tsunami sand layer being sandwiched with sharp contacts with overlying and underlying mud layers, (c) absence of any primary physical or biological structure, and (d) lack of sorting within the layer. Studies which compared storm and tsunami deposits from a variety of environments often link these signatures to a tsunami or an unlikely large storm event (Supplementary Table 2).

The Gonu 2007 super cyclone is considered as the strongest storm in the history of the Arabian Sea, that had impacted the Oman and Iranian coastlines with maximum breaking wave heights of 5.4 m and inundation of 11 m^32^. Some modeling studies suggest that the strongest storm in the Arabian Sea can have wave heights of 9.4 m at a breaking point and inundation of 25 m^33^. Although the Arabian sea experiences several tropical cyclones annually, the strength of these cyclones has been significantly less compared to cyclones in the Pacific and other parts of the world^32–34^. Historically, in the Arabian Sea, the most recent tsunami was the 1945 Makran tsunami which led to a huge loss of human life and destruction along the coastlines of Oman, Iran, Pakistan and India. It had a wave height of 4–5 m in Pasni (Iran), 1.5 m in Karachi (Pakistan) and 2 m in Mumbai (India)^35^. The only available information of wave height^18^ during 1945 Makran tsunami along the Kachchh coast (present study site), is contested^9,10^. The historical record of tsunamis in the Arabian Sea dates back to 326 BC^36^, but is still fragmented. Ambraseys and Melville^35^ reported that during AD 1008, an earthquake and tsunami sunk many ships and killed a lot of people. The epicenter of this earthquake is debated, with some believing it to be in the Persian Gulf ^35^ and some ascribing it to the western part of MSZ^12,14^. This is because, the tectonic boundaries in the Persian Gulf do not seem capable of producing such tsunamigenic earthquakes, whereas they are common in subduction boundaries such as the MSZ. Although the AD 1008 event remains *an enigma* due to variety of reports, Shah-Hosseini *et al*.^6^ reported large coastal boulders from the Iran coastline. Based on ^14^C radiometric ages of shells on the boulders, they postulated that the origin of these boulders is offshore and their deposition in the landward region is due to the AD 1008 tsunami event. They explained the movement of these boulders by a tsunami wave with a 4 m height along the Iranian coastline. Similarly, Hoffmann^37^ reported a cluster of boulders and archeological pottery artifacts from the shoreline of Muscat, Oman, dating back to a tsunami around 1000 years ago. Based on boulder deposits and archeological artifacts, they reported a tsunami event correlated to the 1008 AD event, with a run up of 15 m, which by far exceeds the inundation during the 1945 Makran tsunami event^37^. We believe that in such an event, the tsunami wave would have had a definitive impact on the Indian coastline, Kachchh in particular, due to its strategic geographic position (Fig. 1a).

We, therefore, infer that the sand layer observed in PJ, PG, BO, Mundra, LN and Bhadreshwar has been deposited by the same event, which dates around AD 1008 (Between AD 700 to AD 1460). The origin of this sand layer at different sites was presumably the same and hinting at a ‘sand sheet’ like deposition. The sedimentology of the layer shows its origin to be offshore, with an event that may have eroded the substrate in the offshore region. The tropical cyclones lack the capability to erode the offshore subtidal depths of near coastal regions, compared to tsunami waves which essentially erode the substrate of the offshore environment^28,38,39^. The presence of mud balls and rip up clasts are testimony to an event, which may have eroded the muddy substrate in the offshore region along with subtidal sands^16,34^. The broken shell fragments and shells often point to a high energy hydrodynamic event, which is again reflected in the poor sorting of the sand layer^28,40,41^.

In order to validate the landward extent of this sand sheet, pits were dug inland at a distance >250 m (Fig. 3) from the main sites i.e. PG, BO and LN (baring PJ, because the width of the shoreline is too narrow). It is believed that it is possible to distinguish the storm surge and the tsunami deposits at the hands of facies and lateral extension^28–30,42^. In the present case, the sand sheet has been identified at a distance >200 m inland at PG, BO, LN and Mundra. The sedimentological character of the sand sheet suggests landward fining and thinning of the deposit (Fig. 3), which is consistent with the models of tsunami wave transport^22,28,29,40^.

Based on the present findings, we can surmise that the MSZ has experienced four major tsunamigenic earthquakes during the historical period viz., (1) 4000 year old tsunami inundation with a 15 m run-up at Muscat, Oman coastline^24^, (2) 326 BC tsunami event experienced near present day Karachi, Pakistan^36^, (3) AD 1008 event experienced at the Iranian, Oman and Indian (present day) coastlines ^present study^^6,35^ and (4) AD 1945 event experienced at Oman, Iran, Pakistan and Indian coastlines^7,8,22,23,35^.

Nevertheless, the other causal mechanisms for deposition of this 1000 year old sand sheet, like meteotsunami, reworking of sand from sand bars accreted offshore, cannot be discounted. However, these mechanisms have not been adequately investigated so far and remain elusive. The efforts to explore more palaeotsunami deposits from the coastal archives of India, Iran, Oman and Pakistan should be increased, so as to improve the catalogue of tsunamigenic earthquakes originating from the MSZ. Our findings also have implications on the ongoing worldwide palaeotsunami research as well as the hazard emanating from the MSZ, since it is a likely source of future tsunamis, in this world’s one of the most rapidly developing region. Our report of AD 1008 event from Indian coastline, also supports the claim that the Western MSZ, even if at longer intervals, has experienced major thrust earthquakes (*M*_w_ > 8) in the historical past, as suggested by Heidarzadeh *et al*.^12,43^.

## Methods

Four shallow trenches were dug and one short core was collected from the Kachchh coastline during 2015–2016. The elevation of each trench/core site was measured using high resolution topographic maps and GPS measurements. The sedimentary layers were identified on the basis of physical characteristics and grain size of each unit measured following the standard techniques^44^. The geochemical analysis was carried out with dried and crushed samples using XEPOS HE XRF, at the Institute of Seismological Research (ISR), India. Representative samples from each layer from all the trenches/cores were measured three times in replicate, which gave an analytical precision of <5% for major and trace elemental concentrations. The AMS ^14^C analysis was performed on shells and calibrated using CALIB 7.1 programme^45^. The marine reservoir effect was incorporated using the ΔR value as −8 ± 37^46^. A total of four samples were analyzed at the Poznan Radiocarbon Laboratory, Poznan and Ion beam physics lab, ETH, Switzerland (For details see supplementary data). The OSL dating on three samples was performed at the ISR, India. The collected sand pipes were analyzed in subdued light conditions and palaeodose estimation done following the standard protocol (For details see supplementary data).

## Electronic supplementary material

Supplementary information
