# Supplementary material for: Geological evidence for AD 1008 tsunami along the Kachchh coast, Western India: Implications for hazard along the Makran Subduction Zone
Source: Sci Rep. 2018 Nov 14;8:16816. doi: 10.1038/s41598-018-35193-x (PMC6235955; doi:10.1038/s41598-018-35193-x)
Supplement: Supplementary file 1 — Supplementary information [file 41598_2018_35193_MOESM1_ESM.docx]

**Geological evidence for AD 1008 tsunami along the Kachchh coast, Western India: Implications for hazard along the Makran Subduction Zone**

S. P. Prizomwala^1*^, Drasti Gandhi^1^, Nilesh Bhatt^2^, Wilfried Winkler^3^, M. Ravi Kumar^1^, Nisarg Makwana^1^, Nishith Bhatt^4^

^1^ Active Tectonics Group, Institute of Seismological Research, Gandhinagar, India

^2^ Department of Geology, The M. S. University of Baroda, Vadodara, India

^3^ Geological Institute, ETH-Zentrum, CH-8092 Zürich, Switzerland

^4^ Department of Geology, M. G. Science Institute, Ahmedabad, India

Corresponding author: S. P. Prizomwala

Email: siddharth_prizomwala@yahoo.co.in; prizomwala@isr.res.in

**Coastal Geomorphology**


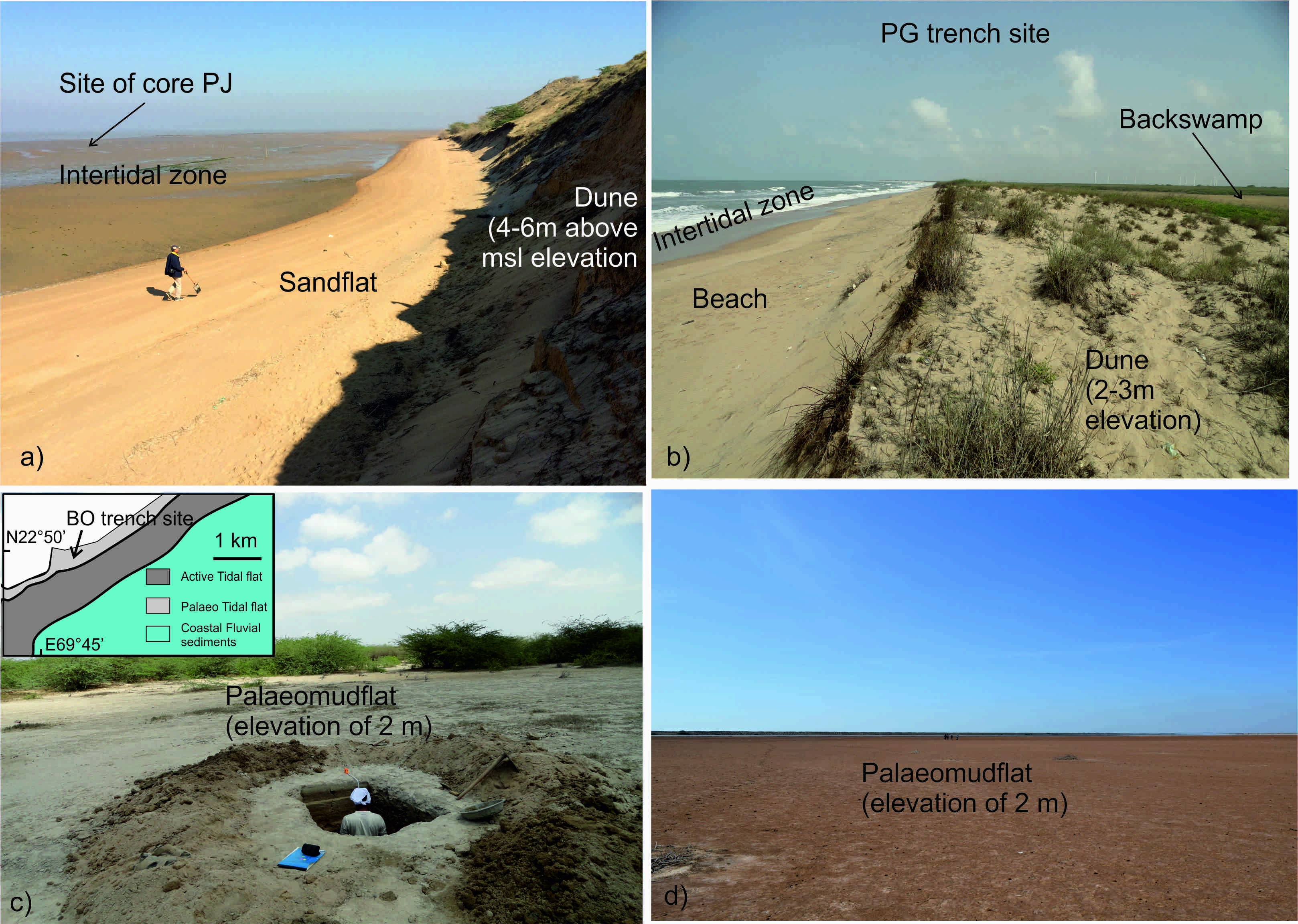


Supplementary figure 1: Coastal geomorphology and view of trench/core site in field photograph a) PJ core site in intertidal zone, b) PG trench in backswamp, c) BO trench in palaeomudflat (insat image shows geomorphic map of the segment) and d) LN trench site setting of palaeomudflat. (Al photographs were taken by SPP)

The Panjorpir (PJ) site is located in the intertidal zone of the southern tip of Kori creek. The coastal landscape (Supplementary fig 1a) shows presence of a 4-6 m high coastal dune field which is as wide as 400 m inland. This is fringed by the narrow sandflat of 10-30 m width, which merges into the intertidal zone capped by a thin layer of sand below which is the mudflat. A shallow core was raised from the intertidal zone at a distance of 200m in seaward direction from the high water line during the low tide period (Supplementary fig 1a). The site of core is within the intertidal zone and hence at 0 m from the msl.

Pingleshwar (PG) site is located in the western part of Kachchh coastline, where a beach-dune-ridge complex camouflages the coastal landscape. The beach is narrow with width not exceeding 15 m, with a steep gradient. The beach ridge-dune runs parallel to the beach with an average elevation of 2-3m above the msl (Supplementary fig 1b). The beach ridge-dune is fringed by the backswamp of 300-400 m width on the landward side, which shows a characteristic typical palaeomudflat like sediment. A shallow trench was dug at a distance of 210m from the high water line.

The Borai (BO) site is located on a palaeomudflat at an elevation of 2m above msl. The gradient is negligible and the segment from Mandvi to Kandla is characterized by this monotonous mudflat configuration. The intertidal zone is hundreds of meters wide and fringed by a palaeomudflat which is at an average elevation of 2-3 m from the high water line near Mundra and the elevation increases towards the eastern end of the Kachchh coastline near Kandla. A trench of 1.5m was dug at the palaeomudflat near Borai (BO site) (Supplementary fig 1c). Similarly, the Luni (LN) site lies in the same segment, 25 km east of Mundra, on a palaeomudflat with an elevation of 2m above the msl (Supplementary fig 1d).

***Grain size analysis***

We performed grain size analysis of the sand horizon of interest in all the trenches/cores and analysed the results together with a few published data from the beach, intertidal and dune environments of Kachchh coast. The grain size analysis was carried out following the conventional Folk^1^ method. Limited samples were considered for grain size analysis, since the main objective was only to characterize the sand layers for their sedimentological character. The landward fining of the sand layer in extension pits/trenches (Fig 3 and supplementary table 1) showed that the sand was mainly coarse grained, with poor sorting and illustrated a bi-model particle size distribution (Fig. 2), which hints at a higher hydrodynamic regime mostly favoured in extreme wave events. In addition to characterization of sand layers, a few samples from known dune, beach and intertidal environments were analyzed and compared with published results, with an aim to emphasize the relative sorting involved in the processes (Supplementary figure 2).

Supplementary Table 1: Grain size distribution of sand layer in various core/pits of Kachchh coast along with some dune, beach and intertidal environment samples following the method of Folk^1^

| **Sample No** | **Median** | **Mean** | **Standard Deviation** | **Skewness** | **Kurtosis** | **Description** |
| --- | --- | --- | --- | --- | --- | --- |
| PJ1 | 2.87 | 2.84 | 1.09 | -0.3 | 1.68 | Poorly Sorted |
| PJ2 | 3.01 | 2.84 | 1.29 | -0.47 | 1.81 | Poorly Sorted |
| PG (Extn trench) | -0.63 | -0.45 | 1.17 | 0.26 | 0.86 | Poorly Sorted |
| PG | -0.12 | 0.71 | 2.02 | 0.47 | 0.92 | Very Poorly Sorted |
| BO (M2) | 0.62 | 0.85 | 2.11 | 0.14 | 0.69 | Very Poorly Sorted |
| BO (M3) | 1.12 | 1.06 | 1.26 | -0.08 | 0.92 | Very Poorly Sorted |
| BO (M4) | 1.18 | 1.2 | 1.12 | -0.02 | 1.01 | Very Poorly Sorted |
| BO (M5) | 1.23 | 1.32 | 1.04 | -0.14 | 1.13 | Very Poorly Sorted |
| LN | 3.42 | 2.48 | 1.65 | -0.76 | 0.69 | Poorly Sorted |
| LN2 | 3.57 | 3.15 | 1.06 | -0.72 | 2.33 | Poorly Sorted |
| LN3 | 3.6 | 3.2 | 0.9 | -0.7 | 2.5 | Poorly Sorted |
| LN dune | 1.45 | 1.43 | 0.59 | -0.04 | 1.29 | Moderately well Sorted |
| LN dune | 1.16 | 1.08 | 0.66 | -0.2 | 1 | Moderately well Sorted |
| LN river 2 | 0.57 | 0.5 | 0.88 | -0.15 | 0.95 | Moderately Sorted |
| LN river 1 | 1.19 | 1 | 1.29 | -0.21 | 1.04 | Poorly Sorted |
| PG intertidal | 0.91 | 0.9 | 0.68 | -0.05 | 0.98 | Moderately Sorted |
| PG beach | 0.67 | 0.75 | 0.66 | 0.18 | 1.16 | Moderately Sorted |
| PG dune | 0.71 | 0.79 | 0.6 | 0.2 | 1.06 | Moderately well Sorted |
| BO beach | 2.43 | 2.47 | 0.46 | -0.03 | 1.43 | Moderately Sorted |

***
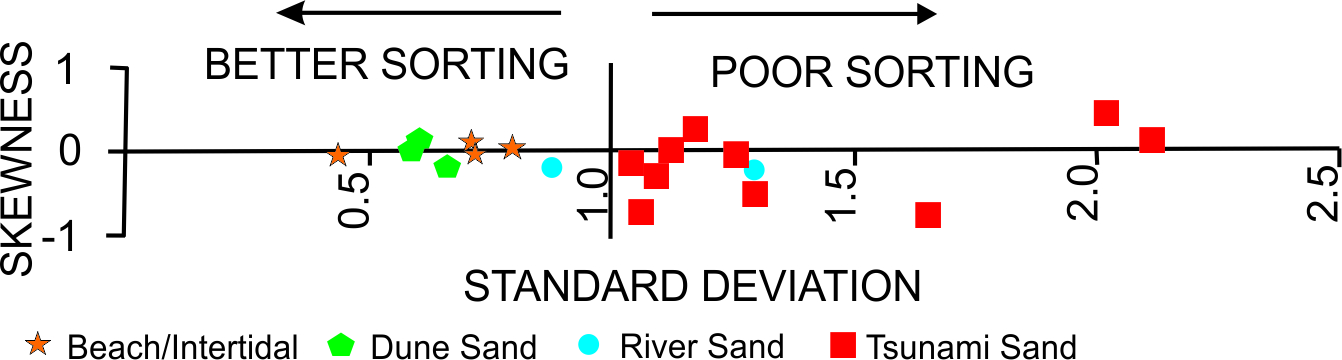
***

Supplementary figure 2: Plot of standard deviation versus Skewness of coastal samples from present study and published work^19,25^

***Chronology***

*OSL dating*

The Optically Simulated Luminescence (OSL) dating of sediments provides the age of the most recent burial event. The method relies on the premise that daylight exposure during the transport and burial, photo-bleaches the geological luminescence to near zero residual level^2^. After burial, the daylight exposure ceases and a re-accumulation of luminescence occurs due to radiation from the decay of ambient natural radioactivity i.e. Uranium (U), Thorium (Th) and Potassium (K). The burial age is calculated as the ratio of acquired luminescence to the rate of luminescence acquisition i.e. radiation dose rate ^2,3^.

The sediments in stainless steel pipes collected from the sand layers were analyzed in subdued light conditions, at the Institute of Seismological Research, India. The middle part of the samples in pipes (unexposed) was pretreated with 10% HCl to remove carbonate and Hydrogen peroxide to remove the organic matter. This was followed by sieving to obtain 90-150 μm grain size fraction. A Frantz magnetic separator (Model-LB-1) was used to remove magnetic mineral assemblages and feldspar grains from pure Quartz grains. The extracted quartz is then treated with 40% HF acid for 80 min followed by concentrated HCl to remove the outer alpha irradiated skin and dissolved residual feldspars. The purity of the extracted quartz in respect of feldspar contamination was checked using IRSL for 100s at room temperature. An automated Risoe TL/OSL reader (TL-DA-15) was used for OSL measurements using blue LEDs emitting at 470 nm and the detection optics comprised of 2 x U-340 and BG-39 filters. Irradiations were made using a 25 mCi^90^Sr/^90^Y beta source. Palaeodose estimates were made using the conventional Single Aliquot Regeneration (SAR) protocol^4^. The OSL measurement was carried out at 125˚C for 40s. The dose recovery test and recycling ratio were used to check the reliability of SAR when applied to our samples. Aliquots with a recycling ratio falling outside 1.0 ± 0.1 were discarded. The cosmic ray dose is calculated following the method suggested by Prescott and Hutton^5^. The concentrations of Uranium, Thorium and Potassium were measured using X-ray fluorescence, at the Institute of Seismological Research, India. A total of 3 OSL ages are estimated from sand layers of PJ, PG and BO (Table 1).

Based on De scatter, the samples with over dispersion > 40 were analyzed with minimum age model, whereas for the samples with over dispersion < 40, weighted mean model was used^6^. Owing to the extreme hydrological conditions during the tsunami event, the lack of bleaching is a concern which yields over estimates in ages. To avoid this for such samples with OD > 40, only minimum De were used and ages estimated (Table 1).

*AMS ^14^C dating*

Additionally, we used AMS ^14^C carbon dating using foraminifers and bivalve shells to estimate the ages of LN and BO sections. The analysis was performed at Poznan Radiocarbon Laboratory, Poznan and Ion beam physics lab, ETH, Switzerland. Few bivalve shells from base of unit-5 sand layer in BO section along with foraminifers from unit-2 were used to estimate the age of the BO section. From the adjacent LN section we picked a few foraminifers from unit-2 and unit-6, which would bracket the age of the suspected palaeotsunami deposit. We used a marine reservoir effect (ΔR) of -8 ± 37 from adjacent Pirotan island in the Gulf of Kachchh^7^. This ΔR is considered to be more accurate, being closest to the studied site and within the gulf, compared to earlier values obtained from the northern Arabian sea using samples from an open sea environment^7^.We used CALIB 7.1 programme for Marine13 dataset^8^ (Table 2). The calibrated ages are expressed as calendar years over a 2σ-error range (95.4%).

***Sedimentary character comparison***

Table 3: Comparison of physical characteristics of sand layer along the Kachchh coastline with typical storm and tsunami deposits^9-18^

|  | | **AD 1008 tsunami event along the Kachchh coastline, Western India** | | | | **2004 Indian Ocean Tsunami** | | **2013 Typhoon Haiyan storm surge** | **2003 Hurricane Isabel western Atlantic Ocean** |
| --- | --- | --- | --- | --- | --- | --- | --- | --- | --- |
| **Site configuration** | *Locality* | *Panjor pir (Pj)* | *Pingleshwar (PG)* | *Borai (BO)* | *Luni (LN)* | *Banda Aceh, Indonesia* | *Kalpakkam and Nagipattinam* | *Basey, Samar* | *Hatteras Island, North Carolina* |
|  | *Coastal geomorphology* | Intertidal zone / mudflat in Kori creek | Backswamp | Palaeomudflat | Palaeomudflat | Narrow beaches bounded by headland | Narrow beaches with coastal dunes | Narrow sandy beach on carbonate platform | Barrier island |
|  | *Elevation from Mean Sea Level (m)* | 0 | 4 | 2 | 2 | 4-35 | < 5 | 2 - 3 | 2 |
| **Sedimentary texture and structure** | *Thickness (cm)* | 32 | 24 | 36 | 34 | 5 - 20 | 10 - 40 | 2 - 8 | 40 - 97 |
|  | *Vertical grading?* | No grading | No grading | No grading, up section increase in shell amount | Fining upward and coarsening upward in two units | Massive units with normal grading | Massive but graded, coarsening to fining upward | No analysis | Normal graded to inverse graded at some places |
|  | *Landward grading* | - | Landward fining | Landward fining | Landward fining | Landward fining | Landward fining | Overall landward fining | - |
|  | *Basal contact* | Sharp erosional | Sharp erosional | Sharp erosional | Sharp erosional | Sharp minimal erosion | Sharp | Sharp depositional | Abrupt with underlying soil |
|  | *Sedimentary structure* | Absent | Absent | Absent | Massive to horizontal | Planer with one section of cross stratification | Planer, massive, microbars and cross lamination | Massive | Planer laminations |
|  | *Sorting* | Poor | Poor | poor | Poor to moderate | Poorly sorted | Poor to moderate | Poorly sorted | Well sorted |
|  | *Landward extent (m)* | - | >220 | > 250 | > 230 | 400 | 150 - 350 | 350 | 200 |
|  | *Longshore extent (Km)* | > 250 | | | | - | >300 | - | - |
|  | *Rip-up clasts* | Absent | Absent | Present | Present | - | - | Absent | Absent |
|  | *Number of layers* | 1 | 1 | 1 | 3 | 1-3 | 2 | - | 7 - 20 |
|  | *Reference* | This study | This study | This study | This study | Moore et al., 2006 | Srinivasalu et al., 2007; Switzer et al., 2012 | Soria et al. 2017 | Morton et al., 2007 |

***Provenance: Geochemical signatures***

***Panjor pir (PJ)***

The geochemical analysis clearly shows a distinct provenance for the Unit-2, i.e. sand layer compared to clayey silt and top sand layers. The unit-2 sand layer is enriched in FeO and TiO2 along with depletion in Sr concentrations. How the same unit is enriched in Rb concentrations whereas unit-4 sand at the top is found to be depleted, is an issue. The geochemistry indicates that the source of the top sand layer is fluvial flux brought by hinterland rivers which drain through the Deccan Trap basalt, and hence rich in Sr and depleted in Rb^19^. The northern Arabian Sea sediments show enrichment of Rb^20^, which is reflected in unit-2 sand layer, which testifies the link of this sand layer to an offshore source.

***Pingleshwar (PG)***

The temporal variations in major oxides (FeO, TiO_2_ and CaCO_3_) and selected trace elemental concentrations (Sr, Zr and Cr) in the Pingleshwar sequence shows that the sand layer (Unit-2) shows depletion in major oxides and trace elements concentrations. The concentration of CaCO_3_ shows an increase in Unit-2, which is most likely due to presence of broken shell fragments. Similarly, the increase in Sr and Zr hints at the sand being originally routed from hinterland provenance to offshore regime, which was then eroded by the extreme wave and deposited in this backswamp region.

***Borai (BO)***

The temporal variations in major oxides (Fe_2_O_3_, TiO_2_ and CaCO_3_) and selected trace elemental concentrations (Sr, Zr and Cr) in the BO sequence reveal that the sand layers (Unit-3 and Unit-5) are depleted in major oxides and trace element concentrations. The concentration of CaCO_3_ shows an increase in Unit-5 and a decrease in Unit-3. This is most likely due to the presence of broken shell fragments in Unit-5 and their lack in Unit-3. We presume that the deposition of Unit-5 is due to a palaeo-tsunami wave, which reworked the shell/bioclastic rich seabed of the Gulf of Kachchh. Hashmi^21^ studied the sediments from the floor of the Gulf of Kachchh and reported that the central and western parts are rich in shell/bioclastic fragments. On the contrary, Unit-3 was deposited on account of a ebb-tidal storm surge, which characteristically would deposit sediments from 1) near shore/intertidal origin and/or 2) landward eroded material while moving back, which in any case would be depleted in shell fragments, hence showing negligible or no shell content. Similarly, concentrations of Zr and Cr have been found to be enriched in marine origin sand layers and have been used as indicators of marine flooding^12,22-24^. Parallel to low CaCO_3_ concentrations, Unit-3 shows lower amounts of Zr and Cr. The higher amount of Zr and Cr in Unit-5 testifies its origin from offshore sands, which are normally rich in these elements compared to fluvial sands of Kachchh. Unit-5 shows enrichment in Sr concentrations compared to Unit-3. The Sr concentrations in Unit-5 are comparable to Sr concentrations in the clayey silt units, which underlines their similar provenance.

***Luni (LN)***

The enrichment in Zr and Cr in units 3 and 5 suggests an offshore origin, which is also supported by an increase in CaCO_3,_ which most likely is governed by the presence of marine shells. The unit-3 seems more linked to an offshore origin, whereas units 5 is more linked to backwash of a retreating wave, as hinted by enrichment of Fe, Ti and Sr, along with depletion in CaCO_3_ in unit 5 compared to unit 3.

References

1. Folk, R.L. The distinction between grain size and mineral composition in sedimentary-rock nomenclature. *Journal of Geology*, **62**, 344-359 (1954).
2. Aitken MJ (1998) An Introduction to Optical Dating. Oxford University press, Oxford.
3. Singhvi AK, Bluszcz A, Bateman MD, Someshwar Rao M (2001) Luminescence dating of loess-palaeosol sequences and coversands: methodological aspects and palaeoclimatic implications. Earth Science Reviews 54: 193–211.
4. Murray AS, Wintle AG (2000) Luminescence dating of quartz using an improved single aliquot regenerative dose protocol. Radiation Measurements 32: 57–73.
5. Prescott JR, Hutton JT (1994) Cosmic ray contributions to dose rates for luminescence and ESR dating: large depths and long term variations. Radiation Measurement 23: 497–500.
6. Galbraith, R.F., Roberts, R.G., Laslett, G.M., Yoshida, H., Olley, J.M., 1999. Optical dating of single and multiple grains from Jimnium rock shelter, northern Australia: part 1, experimemtal design and statistical methods. Archaeometry 41, 339–364.
7. Dutta, K., Bhushan, R., Somayajulu, B. Delta R correction values for the northern Indian Ocean. *Radiocarbon* **43 (2A)**, 483–488 (2001).
8. Stuiver, M., Reimer, P.J., and Reimer, R.W., 2018, CALIB 7.1 [WWW program] at http://calib.org, accessed 2018-8-13
9. Tuttle, M.P., Ruffman, A., Anderson, T. and Jeter, H. Distinguishing tsunami from storm deposits in eastern North America: the 1929 Grand Banks tsunami versus the 1991 Halloween storm. *Seismological Research Letters*. **75(1)**, 117-131 (2004).
10. Morton, R.A., Gelfenbaum, G. and Jaffe, B.E. Physical criteria for distinguishing sandy tsunami and storm deposits using modern examples. *Sedimentary Geology*. **200(3),** 184-207 (2007).
11. Choowong, M., Murakoshi, N., Hisada, K.I., Charusiri, P., Charoentitirat, T., Chutakositkanon, V., Jankaew, K., Kanjanapayont, P. and Phantuwongraj, S. 2004 Indian Ocean tsunami inflow and outflow at Phuket, Thailand. *Marine Geology*. **248(3)**, 179-192 (2008).
12. Ramírez-Herrera, M.T., Lagos, M., Hutchinson, I., Kostoglodov, V., Machain, M.L., Caballero, M., Goguitchaichvili, A., Aguilar, B., Chagué-Goff, C., Goff, J. and Ruiz-Fernández, A.C. Extreme wave deposits on the Pacific coast of Mexico: Tsunamis or storms?—A multi-proxy approach. *Geomorphology*. **139**, 360-371 (2012).
13. Phantuwongraj, S. and Choowong, M. Tsunamis versus storm deposits from Thailand. *Natural Hazards*. **63(1)**, 31-50 (2012).
14. Gouramanis, C., Switzer, A.D., Jankaew, K., Bristow, C.S., Pham, D.T. and Ildefonso, S.R. High-frequency Coastal Overwash Deposits from Phra Thong Island, Thailand. *Scientific Reports*. 7 (2017).
15. Moore, A., Nishimura, Y., Gelfenbaum, G., Kamataki, T., Triyono, R., 2006. Sedimentary deposits of the 26 December 2004 tsunami on the northwest coast of Aceh, Indonesia. Earth, Planets and Space 58, 253–258.
16. Srinivasalu, S., Thangadurai, N., Switzer, A.D., Ram Mohan, V., Ayyamperumal, T., 2007. Erosion and sedimentation in Kalpakkam (N Tamil Nadu, India) from the 26th December 2004 tsunami. Marine Geology 240, 65–75.
17. Switzer, A.D., Srinivasalu, S., Thangadurai, N., Ram Mohan, V., 2012. Bedding structures in Indian tsunami deposits that provide clues to the dynamics of tsunami inundation. In: Terry, J.P., Goff, J. (Eds.), Natural Hazards in the Asia–Paciﬁc Region: Recent Advances and Emerging Concepts. Geological Society, London, Special Publications 361, pp. 61–77.
18. Soria, J.L.A., Switzer, A.D., Pilarczyk, J.E., Siringan, F.P., Khan, N.S., Fritz, H.M. Typhoon Haiyan overwash sediments from Leyte Gulf coastlines show local spatial variations with hybrid storm and tsunami signatures. *Sedimentary Geology*, **358,** 121-138 (2017)
19. Prizomwala, S.P., Bhatt, N. and Basavaiah, N. Provenance discrimination and source-to-sink studies from a dryland fluvial regime: An example from Kachchh, western India. *International Journal of Sediment Research.* **29(1)**, 99-109 (2014).
20. Ramaswamy. V., Nath, B.N., Vethamony, P., and Illangovan, D. Source and dispersal of suspended sediment in the macro-tidal Gulf of Kachchh. *Marine Pollution Bulletin,* **54,** 708-719 (2007).
21. Hashimi N.H., Nair R.R., Kidwai R.M., Sediments of Gulf of Kachchh- A high energy tide dominated environment; *Indian Journal of Marine Sciences* **7** 1-7 (1978).
22. Chagué-Goff, C., 2010. Chemical signatures of palaeotsunamis: a forgotten proxy? Marine Geology 271, 67–71.
23. Goff, J., Pearce, S., Nichol, S.L., Chagué-Goff, C., Horrocks, M., Strotz, L., 2010. Multi-proxy records of regionally-sourced tsunamis, New Zealand. Geomorphology 118, 369–382.
24. Chagué-Goff, C., Goff, J.R., Dominey-Howes, D., Nott, J., Sloss, C., Shaw, W., Law, L., 2011. Tropical Cyclone Yasi and its predecessors. Australian Tsunami Research Centre Miscellaneous Report No. 5, 16 pp. http://www.nhrl.unsw.edu.au.
25. Prizomwala, S.P., Shukla, S.B., Basavaiah, N. and Bhatt, N. Provenance discrimination studies on sediments of the SW Kachchh coast, western India: Insights from heavy mineral and mineral magnetic analysis.  *Journal of Coastal Research.* **29(1)**, 52-60 (2013).
